# Clinical feature of infertile men carrying balanced translocations involving chromosome 10: Case series and a review of the literature

**DOI:** 10.1097/MD.0000000000010452

**Published:** 2018-04-13

**Authors:** Hongguo Zhang, Ruixue Wang, Leilei Li, Yuting Jiang, Han Zhang, Ruizhi Liu

**Affiliations:** a Center for Reproductive Medicine and Center for Prenatal Diagnosis, First Hospital; b Jilin Engineering Research Center for Reproductive Medicine and Genetics, Jilin University, Changchun, China.

**Keywords:** balanced translocation, chromosome 10, chromosome breakpoint, genetic counseling, male infertility

## Abstract

**Rationale:**

Infertile male carrying balanced translocations can be broadly divided into two types: pregestational and gestational infertility. Chromosome and breakpoints involved translocation should be considered in genetic counselling for these patients. To date, > 100 cases have been described with carrying balanced translocations involving chromosome 10 in fertile male.

**Patient concerns:**

We report 11 cases translocation carriers involving chromosome 10, and review 99 carriers of chromosome 10 translocation from reported literature.

**Diagnoses:**

Eleven cases of chromosomal translocation were diagnosed by cytogenetic analysis. Three of these men had azoospermia or oligozoospermia, while eight had normal semen. Of these latter cases, their partners were able to conceive, but had a tendency to miscarry or have a stillbirth.

**Interventions:**

Chromosome breakpoints should be considered in genetic counseling. Preimplantation genetic diagnosis should be performed to decrease the high risk of miscarriage and to minimize the genetic risks to offspring for patients with gestational infertility.

**Outcomes:**

The most common translocations and breakpoints were at t(4;10) and 10q24, observed in 12 and 10 patients respectively. Breakpoints at 10p15.1, 10p12, 10q10, 10q22.1, 10q24.2, and 10q26.3 were linked to pregestational infertility; breakpoints at 10p12.1, 10q11, 10q21.2, and 10q23.3 were associated with gestational infertility; the other breakpoints were connected with both forms of infertility.

**Lessons:**

Breakpoints at 10p12 and 10q26.3 were associated with pregestational infertility. Other breakpoints at chromosome 10 were correlated with gestational infertility. These breakpoints should be considered when counseling men with chromosome 10 translocations should be informed of their options.

## 1 Introduction

Chromosomal abnormalities are often identified as a cause of male infertility,^[1]^ and balanced chromosomal translocations are the most common structural rearrangement.^[2]^ Individuals affected by such translocations with failure of spermatogenesis because of meiotic impairment can have gamete formation problems, and these can lead to either recurrent pregnancy loss, or a child with intellectual disability, and/or multiple congenital malformations.^[3]^ These effects are associated with specific chromosomes and breakpoints involved in the translocations.^[4]^ Most breakpoints in balanced chromosome translocations are at, or near segmental duplications,^[3]^ some of which can disrupt the structure of important genes, leading to male infertility.^[5]^

There might be important genes associated with spermatogenesis or sperm function on chromosome 10. For example, meiosis-expressed gene 1 (MEIG1) is located on chromosome 10p13, and is associated with the control of spermiogenesis.^[6]^ Sperm-associated antigen 6 (SPAG6) gene, mapped on chromosome 10 at 10p12.2, is essential for sperm flagellar motility, and is important for the maintenance of the structural integrity of mature spermatozoa.^[7]^ The wings apart-like protein (WAPL) gene is located on chromosome 10q23.2, and is implicated in spermatogenesis.^[8]^ A breakpoint at 10q24 is also linked with impaired spermatogenesis and recurrent spontaneous abortion.^[9]^

Genetic counseling remains a challenge for male carriers of chromosomal translocations. Research has shown that clinical characteristics do not differ between couple with recurrent miscarriages carrying a structural chromosomal abnormality who accept and those who decline preimplantation genetic diagnosis (PGD) after extensive genetic counseling.^[10]^Similarly, a systematic review concluded a lack of sufficient evidence that PGD can improve the live birth rate in couples with such carriers.^[11]^ The live birth rates on the first trial for couples who chose PGD, and the first pregnancy rate for couples who desired natural conception after genetic counseling were between 37.8, to 53.8%, respectively. Although, the time required to achieve pregnancy was similar in both groups, the cumulative live birth rates were between 67.6 to 65.4%, respectively.^[12]^

This study was established to explore the clinical features, and translocation breakpoints in male carriers of reciprocal chromosomal translocations involving chromosome 10. This paper also highlights the importance of genetic counseling, and the option of using assisted reproductive technologies, and PGD for such men who might be infertile.

## 2 Patients and methods

### 2.1 Study design

We performed a single-center retrospective study, and searched literature on translocations in chromosome 10 from infertile men was performed using PubMed, Google Scholar and the China National Knowledge Infrastructure (CNKI) database.

### 2.2 Patient selection

Between the years July 2010 to June 2017, we recruited 11 male carriers (25.9 ± 3.6 years) of chromosome 10 translocations experiencing infertility—or receiving associated counseling—from the outpatient department at the Centre for Reproductive Medicine, the First Hospital of Jilin University, Changchun, P. R. China. All patients underwent a thorough physical examination, semen analyses, and were required to complete a detailed questionnaire concerning their marital status, and reproductive, and medical history. The study protocol was approved by the Ethics Committee of the First Hospital of Jilin University, and written informed consent was obtained from each participant.

### 2.3 Semen analysis

Samples were obtained by masturbation from each patient, after 3 to 7 days of abstinence. Semen analysis was performed according to the World Health Organization guidelines. If no spermatozoa were found, the samples were analyzed again after concentrating them by centrifugation. Azoospermia and oligozoospermia were defined as described.^[5]^ All analyses were performed at the same laboratory, and all data were accessed from medical records.

### 2.4 Cytogenetic analysis

Chromosomes from cultured peripheral blood lymphocytes were analyzed after G (Giemsa)-banding. Peripheral blood samples (0.5 mL) from each patient were collected in sterile tubes containing 30 U/mL heparin. Lymphocytes were cultured for 72 hours at 37°C in appropriate culture medium (Yishengjun; Guangzhou Baidi Biotech, Guangzhou, P. R. China). Colcemid (20 *μ*g/mL) was added to the cultures 1 hour before harvesting. Karyotyping of metaphase chromosomes were performed as described.^[5]^ In all cases, at least 20 metaphase plates were counted, and 6 karyotypes were analyzed for each patient. The resolution level of the chromosome analysis was between 400 to 550 band levels. The karyotype nomenclature was described in accordance with the International System for Human Cytogenetic Nomenclature 2009.

### 2.5 Analysis of identified translocation breakpoints

A search for reports on translocations in chromosome 10 from infertile men was performed using PubMed, Google Scholar and CNKI database. The keywords were “chromosome / translocation / sperm” and “chromosome / translocation / abortion” for these searches. We included cases of balanced chromosomal translocations involving chromosome 10 for adult fertile-age men, and excluded those cases without breakpoints involving chromosome 10, or those with complex chromosomal translocations, chimeras, or live born children. The links of translocation breakpoints with male infertility, and recurrent pregnancy loss were analyzed. A total of 91 carriers of chromosomal 10 translocations were found by these searches.

## 3 Results

Eleven translocation carriers involving chromosome 10 were detected in this study. Karyotype results from these 11 patients are summarized in Table 1. Three of these men had azoospermia or oligozoospermia (i.e., leading to pregestational infertility), while 8 had normal semen. Of these latter cases, their partners were able to conceive, but had a tendency to miscarry, or have a stillbirth (defined as gestational infertility): thus, 5 couples had experienced recurrent spontaneous abortions, and 3 had experienced 2 stillbirths.

**Table 1:**
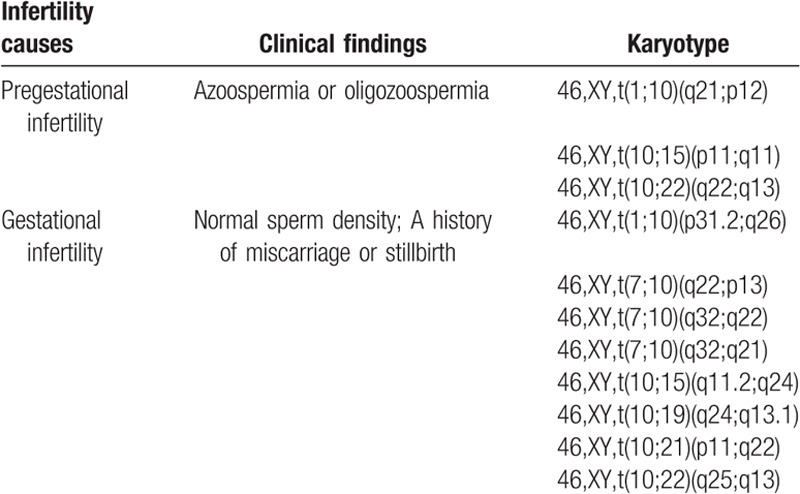
Karyotypes of chromosome 10 translocation carriers and their clinical features.

Karyotyping results, clinical manifestations, and the breakpoints on chromosome 10 from literature analysis were collected, and are shown in Table 2 . The most common translocation was t(4;10), observed in 12 patients, followed by t(7;10) (N = 10), and t(10;11) (N = 8). A total of 101 karyotypes included chromosome 10 translocations. The most common breakpoint, at 10q24, was observed in 10 patients, followed by 10q26 (N = 9) and 10p13 (N = 8). Breakpoints at 10p15.1, 10p12, 10q10, 10q22.1, 10q24.2, and 10q26.3 were linked to pregestational infertility, and breakpoints at 10p12.1, 10q11, 10q21.2 and 10q23.3 were associated with gestational infertility. The other breakpoints were linked to both pregestational and gestational infertility (Table 3).

**Table 2:**
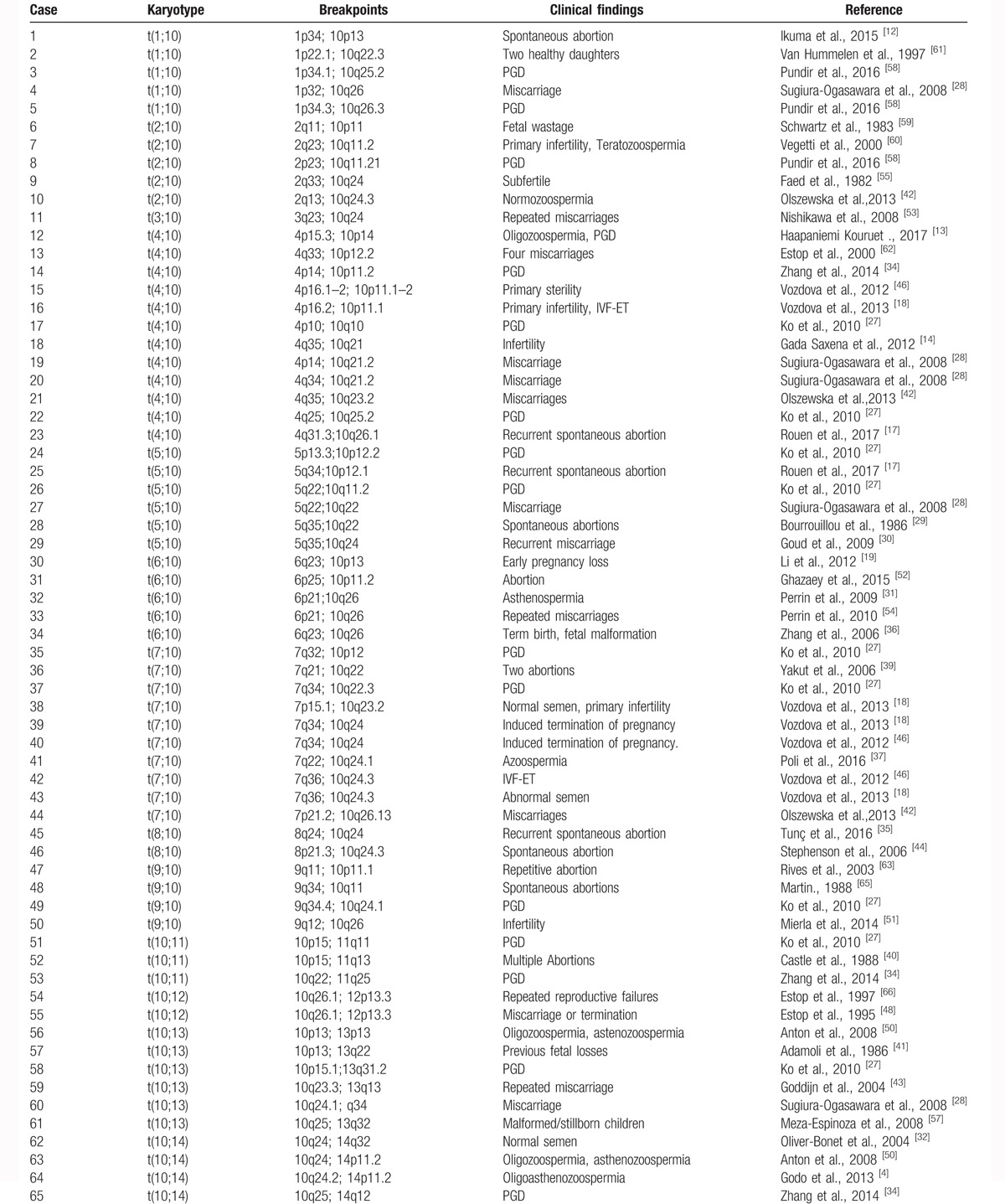
Breakpoints in chromosome 10 translocation carriers and clinical features reported in the literature.

**Table 2 (Continued):**
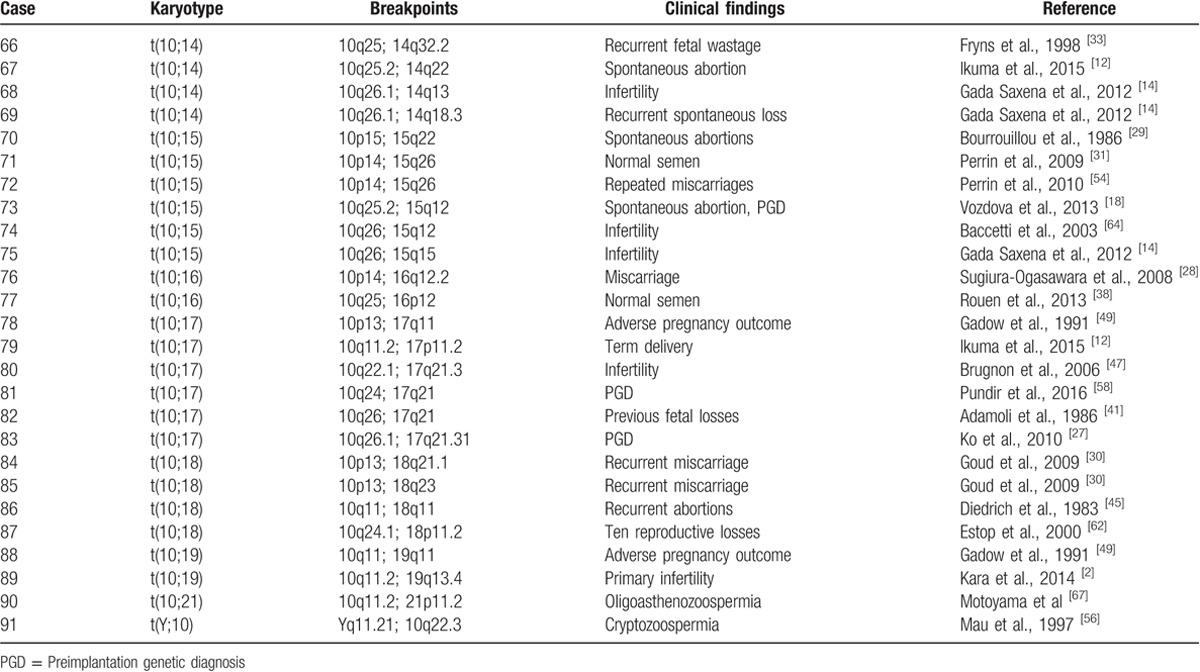
Breakpoints in chromosome 10 translocation carriers and clinical features reported in the literature.

**Table 3:**
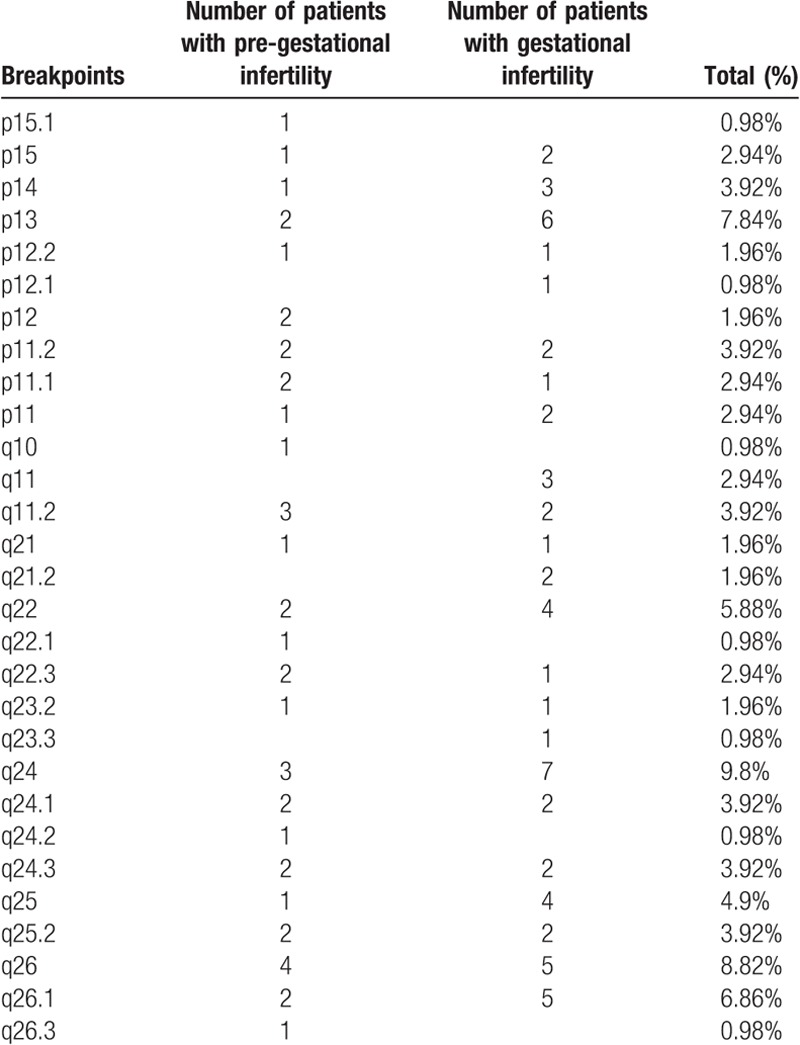
Incidence of breakpoints on chromosome 10.

## 4 Discussion

Many factors are known to cause male infertility. Chromosomal abnormalities are perhaps best known to interfere with spermatogenesis. Reciprocal translocations are the most common among such aberrations.^[13]^ In general, the carriers of balanced chromosomal translocations have a normal phenotype but often have fertility problems, such as infertility, repeated miscarriages, or birth of a child affected with congenital abnormalities.^[14]^ Balanced translocations have a variable influence on the carrier's sperm counts, which can range from normal to low, or even to total aspermia.^[5]^ Hence, genetic counseling for male carriers of translocations remains challenging. Kim et al^[9]^ reported the distributions of chromosomal breakpoints in men with chromosome rearrangements and fertility problems. Manvelyan et al^[15]^ reported that translocations were observed in both partners among these couples, and chromosome 10 was involved as the fourth most frequent to be involved with different chromosomal breakpoints. We have also reported the incidence of breakpoints on chromosome between 2 to 4 in infertile men and have reviewed their impact on genetic counseling.^[2,16]^ In fact, chromosome 10 translocations have often been associated with reproductive failure.^[14,17,18]^ In the present study, 11 of the men in our cases were identified as carriers of chromosome 10 translocations, and 91 cases of chromosome, 10 translocation were also reviewed from the literature.

Reproductive failure is defined as the inability to conceive, or to carry a pregnancy to term, and the 2 most common clinical problems are infertility, and recurrent spontaneous miscarriage.^[14]^ Similarly, male infertility can be broadly, divided into 2 types: pregestational and gestational infertility.^[19]^ Here, the breakpoints that we identified on chromosome 10 were found to be associated with pregestational or gestational infertility. Three cases were associated with pregestational infertility and 8 cases with gestational infertility. Kim et al^[9]^ reported that the breakpoint at 10q24 could interfere with spermatogenesis, and be linked to recurrent abortion. To explore the relationship between these breakpoints on chromosome 10, and male infertility, we analyzed the recent literature, and reviewed the clinical features in carriers of chromosome 10 translocations. The karyotype results, and clinical findings for chromosome 10 are summarized in Table 2 . Recurrent miscarriage was the most common clinical finding in these cases. Translocations involving chromosome between 4 to 10 were the most common, observed in 12 infertile men.

Table 3 shows that breakpoints at 10p15.1, 10p12, 10q10, 10q22.1, 10q24.2, and 10q26.3 were linked to pregestational infertility. The SPAG6 gene mapped on chromosome 10 at 10p12.2 has been reported to be essential for sperm flagellar motility, and important for maintenance of the structural integrity of mature spermatozoa.^[7]^ Synaptonemal complex central element protein 1 (SYCE1) is located on chromosome 10q26.3, and defects in this are associated with spermatogenic failure.^[20]^ For the 10q10 situation involving whole-arm translocation, some have reported that the translocation makes pairing between homologous chromosomes difficult during meiosis, and can lead to segregation defects during spermatogenesis or to oligozoospermia. In addition, epigenetic effects might contribute to the phenotypic defects associated with breakpoints caused by translocations.^[21]^ Other breakpoints were associated with gestational infertility, and some of these were also linked to pregestational infertility. For the latter cases, the breakpoints were not responsible for pregestational infertility, so another breakpoint or translocation might have been involved in these individuals. For example, the breakpoint at 10q24 was the most common finding in our series, and was associated with both pregestational and gestational infertility (Table 3). The clinical features associated with this were consistent with the literature.^[9]^ Additionally, excluding the cases of gestational infertility, pregestational infertility might be linked to another breakpoint at 10q26. The man in case 32 with t(6;10)(6p21;10q26) had asthenospermia, and the man in case 75 with t(10;15)(q26; q15) was infertile. Solute carrier family 26, member 8 (SLC26A8) mapped on chromosome 6 at 6p21 has been reported to be associated with nonobstructive asthenozoospermia.^[22]^ Cation channel, sperm-associated, 2 (CATSPER2) is located on chromosome 15q15.3, and is associated with nonsyndromic male infertility.^[23]^ Further research will be necessary to explore the molecular mechanisms, and genetic basis for these carriers, and their phenotypes. Chromosomal breakpoints and translocations should be considered in genetic counseling. Additionally, some of these reported cases required PGD (Table 3). PGD, and prenatal diagnosis can be performed to decrease the high risk of miscarriage, and to minimize the genetic risks to offspring.^[13]^ However, PGD is expensive, and a lower success rate for PGD is seen for male carriers of chromosomal anomalies, and might be associated with the age of the man, and of his spouse.^[24,25]^ Although the infertility of male carriers of a balanced chromosomal translocation can be explained by the meiotic segregation of the rearrangement, and/or by poor quality semen, further attempts at natural conception remain a viable option for some carriers because of the encouraging reported cumulative live birth rate of 64.3%.^[26]^ For these carriers, informed choice should be offered as discussed below.

In conclusion, a total of 102 carriers of chromosome 10 translocations were reviewed. The most common translocation, and breakpoint was t(4;10), and 10q24, respectively. Breakpoints at 10p12, and 10q26.3 were associated with pregestational infertility. Other breakpoints in chromosome 10 were associated with gestational infertility. These breakpoints should be considered in genetic counseling.

## Author contributions

**Writing – original draft:** Hongguo Zhang.

**Investigation:** Ruixue Wang.

**Methodology:** Leilei Li, Yuting Jiang.

**Writing – review & editing:** Han Zhang, Ruizhi Liu.

**Funding acquisition:** Ruizhi Liu.
